# *MECP2* variation in Rett syndrome—An overview of current coverage of genetic and phenotype data within existing databases

**DOI:** 10.1002/humu.23542

**Published:** 2018-05-21

**Authors:** Gillian S. Townend, Friederike Ehrhart, Henk J. van Kranen, Mark Wilkinson, Annika Jacobsen, Marco Roos, Egon L. Willighagen, David van Enckevort, Chris T. Evelo, Leopold M. G. Curfs

**Affiliations:** ^1^ Rett Expertise Centre Netherlands ‐ GKC Maastricht University Medical Center Maastricht The Netherlands; ^2^ Department of Bioinformatics ‐ BiGCaT NUTRIM Maastricht University Maastricht The Netherlands; ^3^ Institute for Public Health Genomics Maastricht University Maastricht The Netherlands; ^4^ Center for Plant Biotechnology and Genomics Universidad Politécnica de Madrid Madrid Spain; ^5^ Department of Human Genetics Leiden University Medical Center Leiden The Netherlands; ^6^ Department of Genetics University Medical Center Groningen University of Groningen Groningen The Netherlands

**Keywords:** databases, FAIR data, genetic variation, MECP2, phenotype, Rett syndrome

## Abstract

Rett syndrome (RTT) is a monogenic rare disorder that causes severe neurological problems. In most cases, it results from a loss‐of‐function mutation in the gene encoding methyl‐CPG‐binding protein 2 (*MECP2*). Currently, about 900 unique *MECP2* variations (benign and pathogenic) have been identified and it is suspected that the different mutations contribute to different levels of disease severity. For researchers and clinicians, it is important that genotype–phenotype information is available to identify disease‐causing mutations for diagnosis, to aid in clinical management of the disorder, and to provide counseling for parents. In this study, 13 genotype–phenotype databases were surveyed for their general functionality and availability of RTT‐specific *MECP2* variation data. For each database, we investigated findability and interoperability alongside practical user functionality, and type and amount of genetic and phenotype data. The main conclusions are that, as well as being challenging to find these databases and specific *MECP2* variants held within, interoperability is as yet poorly developed and requires effort to search across databases. Nevertheless, we found several thousand online database entries for *MECP2* variations and their associated phenotypes, diagnosis, or predicted variant effects, which is a good starting point for researchers and clinicians who want to provide, annotate, and use the data.

## 1 INTRODUCTION

Rett syndrome (RTT; MIM# 312750) is one of 5,000–8,000 known rare diseases that together have been identified as affecting 6%–8% of the world's population. Approximately 80% of these diseases have a genetic origin (Council Recommendation on an action in the field of rare diseases (2009/C 151/02), Recital 5). Most of these diseases are caused by pathological variants in one single, disease‐specific gene. In the case of RTT, this is in *MECP2*, an important regulator of neuronal development and function (Ehrhart et al., 2016; Lyst & Bird, 2015). At the present time, around 900 unique variations in *MECP2* have been identified (Gold, Krishnarajy, Ellaway, & Christodoulou, 2018). To help distinguish between pathological and neutral genetic variants (Hunter et al., 2016), scientists and clinicians collect genetic data and corresponding phenotypic information and make this information available in databases, which can be used for research and prognostic purposes. In this respect, RTT serves as an example for any monogenic rare disease where, due to the limited number of individuals, a better understanding of the disease can be reached through combining data from different databases that may be housed at different institutions and in different countries. In recent years, the European Union's policy on rare diseases (e.g., Directive 2011/24/EU) has recognized the value of sharing information, knowledge and expertise, and has generated a number of initiatives to encourage pan‐European collaboration, for example, through the creation of European Reference Networks (ERNs) such as Intellectual disability TeleHealth And Congenital Anomalies (ITHACA), the ERN focused on rare congenital malformations and rare intellectual disability in which RTT is placed (https://ec.europa.eu/health/sites/health/files/ern/docs/ernithaca_factsheet_en.pdf).

Generally, there are different types of databases for rare diseases: (1) Patient registries, containing i.a. patient data, genetic data, phenotype descriptions and information on medication. These are not normally open to the public. There are several data platforms, for example, RD‐connect, which host patient registries with controlled access. (2) Genetic data repositories, for example, EGA (European Genome‐Phenome Archive). These have been increasing in number since next‐generation sequencing (NGS), and especially whole exome sequencing (WES), has been used as a clinical standard for the diagnosis of rare disorders and other suspected genetic disorders. (3) Genotype–phenotype databases that combine genetic data (e.g., DNA sequences, variants, genotypes) with phenotypic data. (4) Databases that store general information about genes, proteins, metabolites, their interactions and their mutation specific aberrations.

It is within this context that rare disease registries and databases have also been recognized by the European Union as “key instruments to develop clinical research in the field of rare diseases, to improve patient care and healthcare planning” (https://ec.europa.eu/health/rare_diseases/policy/registries_en).

This study focusses on the genotype–phenotype databases. Several such databases have been developed and will be discussed here. The fundamental goal of these databases is to collect and provide access to data and knowledge to promote research into the functional and pathogenic significance of genetic variants (Brookes & Robinson, 2015; Johnston & Biesecker, 2013). Critical for accurate analysis is the ability to distinguish between the disease‐causing alleles and the abundance of benign variants or less important functional variants that co‐occur in both normal and disease‐affected individuals. One consequence of the increased power of NGS—often used for gene panels, WES, and whole genome sequencing (WGS)—is the increased danger of incorrect assignment of pathogenicity, when compared with single gene analysis. For instance, a typical WES (e.g., in the context of suspected diagnosis of a rare monogenic disorder) may uncover up to 25,000 variants (Gilissen, Hoischen, Brunner, & Veltman, 2012). Elucidation of just a handful of pathogenic variants from the resulting thousands continues to be a major challenge in spite of the availability of standardized software solutions. The most effective way to start distinguishing benign from pathogenic variants is based on population frequencies of variants. In this approach, all variants occurring in the population at higher frequencies than the disease prevalence are considered benign. From the many recent initiatives to collect exome variants of individuals without clear disease phenotypes, the Exome Aggregation Consortium (ExAC) is the largest, containing more than 60,000 exomes (Exome_Aggregation_Consortium, Lek, & MacArthur, 2015). In general, the population frequency information will reduce the number of candidate (pathogenic) mutations to a couple of hundred (Gilissen et al., 2012). Further prioritization can then take place by employing tools such as PolyPhen and SIFT (Sorting Intolerant From Tolerant). Ensembl's Variant Effect Predictor tool (Lelieveld, Veltman, & Gilissen, 2016) makes these aforementioned classic approaches available; it also includes a number of newer methods to distinguish between pathogenic, implicated, associated, damaging, and deleterious variants, and/or those of unknown significance among the remaining variants. These next steps in the prioritization process are summarized by Lelieveld et al. (2016). The challenge of distinguishing disease‐causing sequence variants from the many potentially functional variants in any human genome recently prompted MacArthur et al. (2014) to propose guidelines for investigating causality of sequence variants in human disease. The proper setup and use of databases is one of the key issues they identified in order to be able to upload, store and find pathogenic and benign variants.

The results of the analysis of disease‐causing variants also provides vital information, not just for scientists and researchers who are seeking to further knowledge and understanding of certain diseases, but for clinicians to make the correct diagnosis and provide genetic counseling and patient care. State of the art genotype–phenotype databases are of particular value, and among these, the so‐called locus‐specific mutation databases (LSDBs) (e.g., LOVD (Fokkema et al., 2011)) have served diagnosticians for many years by facilitating the interpretations of genetic variants (Brookes & Robinson, 2015; Johnston & Biesecker, 2013). In addition to the LSDBs, a variety of other (clinically relevant) databases with a focus on genotype–phenotype relationships has emerged in recent years (Lelieveld et al., 2016) and the need to integrate information from these databases has also generated many initiatives. The RD‐Connect project provides a platform for the rare disease community to find and share data and tools (Thompson et al., 2014). It includes a pipeline to harmonize variant annotation of rare disease genomes (Laurie et al., 2016), registries of rare disease registries and biobanks (Gainotti et al., submitted), and bioinformatics tools. It is developed in collaboration with infrastructures such as ELIXIR (https://www.elixir-europe.org/), BBMRI‐ERIC (https://www.bbmri-eric.eu/ (Mayrhofer, Holub, Wutte, & Litton, 2016)), the infrastructure consortium for biobanks, and the Global Alliance for Genomics and Health (GA4GH, https://genomicsandhealth.org). The creation of GA4GH in 2013 represents one of the most prominent large‐scale initiatives in this area. The goals and progress of this group were published recently (GA4GH, 2016)

To support both clinicians and researchers, we present in this article an overview of a number of current genotype–phenotype databases. We evaluate their general structure and function for use in biomedical research, especially for researchers/clinicians who want to find “their” mutation or intend to find a database in which to store their genotype–phenotype data. We give an indication of the findability and interoperability, the practical user functionality (up and download functions), the type and quantity of genotype and phenotype data available, and provide suggestions for future improvement.

## 2 MATERIALS AND METHODS

### 2.1 Selection of databases

The databases and meta/integrated databases in this survey were selected according to the following criteria:

1. The database contains genetic variation and associated phenotypic information (genotype–phenotype databases);
2. The genetic data are available in a processed form to enable a direct search for variations in a specific gene, region, or disease (e.g., in the HGVS or reference SNP (rs) format, an identifier given by the database dbSNP);
3. The database is available online (with or without prior registration);
4. The database is available in English.

We do not claim complete coverage of all available databases; we focus on those which were findable online using search engines (e.g., Google) or listed in http://FairSharing.org (formerly known as BioSharing.org) or other meta‐databases (RD‐connect, bioCADDIE). We evaluated as a separate category certain meta or integrated databases, which in themselves contain no new or unique information, but instead try to integrate information from others. However, a number of RTT‐specific databases, akin to patient registries, were not included in our evaluation as they require membership of the consortium and an agreement to input data to the database, or they grant permission on a case‐by‐case basis when the request to access data is part of a specific research project with prior approval from a medical ethical board. In some instances, a minimal level of data is accessible to qualified researchers through already existing data‐sharing rules. These include the database associated with the longitudinal, population‐based Australian Rett Syndrome Study (AussieRett) (https://rett.telethonkids.org.au/about/aussierett/, (Downs & Leonard, 2013)), the International Rett Syndrome Database (InterRett) (https://rett.telethonkids.org.au/about/interrett/, https://interrett.ichr.uwa.edu.au//output/index.php, (Louise et al., 2009)), the Rett Database Network (https://www.rettdatabasenetwork.org, (Grillo et al., 2012)), and the database generated by the US Rett Syndrome Natural History Study (NHS) (https://www.rettsyndrome.org/research/clinical-trials/natural-history-study) (Neul et al., 2014). These databases generally contain cross‐sectional and longitudinal natural history data that has been directly acquired from or input by individuals and their families, either by families completing a questionnaire or through direct examination of the individual by a clinician experienced in RTT. Such methods of data collection differ from the genotype–phenotype databases of interest in this article.

### 2.2 Assessment of database properties and functions

#### 2.2.1 Aspects of FAIR

The FAIR metrics are not yet fully developed (Schultes et al., in preparation) but as several of these aspects are interesting for the purposes of our evaluation we checked whether each database meets the basic FAIR principles described by Wilkinson et al. (2016). These principles define that data is: (i) *findable* if data or meta data are assigned unique identifiers, described with rich metadata, and registered or indexed in a searchable resource; (ii) *accessible* if the data are retrievable by their identifiers via a standardized communication protocol, the protocol itself is open, free, universally implementable and allows authentication and authorization, whilst, to prevent data being lost, metadata continues to be accessible even when the data is no longer available; (iii) *interoperable* if a suitable language for knowledge presentation and an established vocabulary (e.g., ontologies) are used, and, ideally, the (meta)data include references to other data; and (iv) *reusable* if a clear and accessible data usage license is available, the data are correctly and sufficiently described using domain‐relevant community standards, and data origin and history are included.

#### 2.2.2 Upload and download functions

To investigate user functionality, we looked especially at the upload and download functions of each database. The upload functions were typically found in separate “submit” pages or information was given on how or to whom the data should be sent. For download functionality we checked whether we could manually download search results, for example, a list of *MECP2* variants, and which formats were possible for this. Additionally, we looked for the API description (if available).

#### 2.2.3 Form of genetic and phenotypic data

Each database was investigated for the form in which genetic variation (e.g., HGVS or rs) and phenotype information (e.g., diagnosis, predicted pathogenicity scores, HPO terms etc.) is stored.

### 2.3 Assessment of RTT/*MECP2* specific content

#### 2.3.1 Total numbers of *MECP2* variants in the database

The total number of entries for (unique) *MECP2* variants, or variants which are associated with RTT, was assessed in each database (status March 2018).

#### 2.3.2 Availability of five selected test variants

To examine the coverage of *MECP2* variants in more detail, five *MECP2* mutations were selected and used to perform test searches within each database (Table 1). We decided upon three “classical” variants: first, a well‐known and well‐described mutation—an MBD hotspot mutation—published by Zappella, Meloni, Longo, Hayek, & Renieri (2001) and reviewed by Lyst & Bird (2015); and, second and third respectively, two of the most frequently reported nonsense and missense mutations. Finally, two mutations that were discovered more recently by WES and WGS: a 23 bp deletion in the C‐terminus of *MECP2*, reported by Rauch et al. (2012) after performing WES in a girl displaying a RTT‐phenotype; and, an intra‐exonic deletion, taken from Gilissen et al. (2014), after WGS in a person described as having severe intellectual disability (IQ < 50), a commonly reported clinical phenotype of RTT (Zoghbi, 2016). The appearance of each of these five mutations in the selected databases was investigated.

**Table 1:** *MECP2* mutations selected for test database searches

	Variant 1	Variant 2	Variant 3	Variant 4	Variant 5
**Source**	MBD hotspot mutation from (Zappella et al., 2001)	Frequently reported nonsense mutation	Frequently reported missense mutation	WES variant from (Rauch et al., 2012)	WGS variant from (Gilissen et al., 2014)
**Genomic level** (GRCh37)^a^	g.153296882G>A	g.153296777G>A	g.153296363G>A	g.153296093_153296115del	g.153295929_153296514del
**RNA level (NM_004992.3)**	c.397C>T	c.502C>T	c.916C>T	c.1200_1222del	c.765_1350del
**Protein level**	p.(Arg133Cys)	p.(Arg168^*^)	p.(Arg306Cys)	p.(Pro401Argfs*8)	p.(Lys256Asnfs*31)

a The current genome build at the time of writing this article is GRCh38, but most databases were using GRCh37. For *MECP2*, there is a difference ranging from735 to 659 kbp.

## 3 RESULTS

We identified nine standalone databases and four meta/integrated databases for evaluation (Table 2) and collected information by exploring their content. We checked for general database features and RTT‐specific entries. In detail, we analyzed (a) the FAIR status, (b) the upload and download possibilities, (c) form of phenotype and genotype information, (d) the total number of entries relating to the *MECP2* gene or RTT, and (e) the coverage for the chosen *MECP2* mutations.

**Table 2:** Overview of databases included in the review

Database and link	Contact	How to cite (literature reference for database)	Short description
**RTT‐specific database**			
http://mecp2.chw.edu.au/	about:blank and mailto:rahul.krishnaraj@health.nsw.gov.au	Christodoulou et al. (2003)	Specific focus on RTT. Database of genetic information about RTT patients. Contains mutation information about MECP2 as well as CDKL5 and FOXG1 which cause different syndromes (formerly named Rett‐like syndromes).
**Databases for genetic variations and phenotype information for diseases in general**			
https://kmd.nih.go.kr/kmd/kmd?type=emain https://kmd.nih.go.kr/kmd/search?type=mimid&query=312750 (Korean Mutation Database)	Contact via http://www.cdc.go.kr/CDC/eng/main.jsp (Korea Centre for Disease Control and Prevention)	–	Genotype‐disease database. Collection of disease‐causing variants in genes.
https://www.ncbi.nlm.nih.gov/clinvar/?term=MECP2%5Bgene%5D https://www.ncbi.nlm.nih.gov/clinvar/?term=MECP2%5Bgene%5D	about:blank	Landrum et al. (2016)	Genotype–phenotype database. Focus on disease‐causing variants in genes.
HGMD “professional”	http://www.hgmd.cf.ac.uk/docs/comment_form.html (via public Website)	Stenson et al. (2017)	Commercial genotype–phenotype database
**Databases for all kinds of genetic variations and phenotype information**			
https://databases.lovd.nl/shared/genes/MECP2 http://databases.lovd.nl/shared/genes/MECP2 (Leiden Open Variation Database)	MECP2 curator: mailto:henk.van.kranen@rivm.nl	Fokkema et al. (2011)	Genetic variants database. Locus/gene specific, all genes.
https://decipher.sanger.ac.uk/ (DatabasE of genomiC varIation and Phenotype in Humans using Ensembl Resources)	mailto:decipher@sanger.ac.uk	Firth et al. (2009)	Genotype–phenotype database. All genes.
http://evs.gs.washington.edu/EVS/ http://evs.gs.washington.edu/EVS/ServletManager?variantType=snp&popID=EuropeanAmerican&popID=AfricanAmerican&SNPSummary.x=43&SNPSummary.y=10 (Exome Variant Server)	mailto:evsserver@uw.edu	–	Genetic variants database. Originally those which contribute to heart, lung and blood disorders. Now open to all genes, linked to dbSNP and dbGAP.
http://exac.broadinstitute.org/gene/ENSG00000169057 (Exome Aggregation Consortium)	https://github.com/konradjk/exac_browser/issues mailto:exomeconsortium@gmail.com	Lek et al. (2016)	Database/project to collect and harmonize whole exome sequencing data. Allows search for variants at certain locations or single genes, and direct search for variants.
https://www.ncbi.nlm.nih.gov/snp/?term=MECP2+%5Bgene%5D (NCBI Short Genetic Variations database) https://www.ncbi.nlm.nih.gov/snp/?term=MECP2+%5Bgene%5D	mailto:snp-sub@ncbi.nlm.nih.gov	Kitts et al. (2013)	Genetic variation database. Collection of single nucleotide polymorphism (SNP) and an effect predictor score.
**Integrated/meta‐databases and genome browsers**			
https://www.ncbi.nlm.nih.gov/dbvar/?term=MECP2 dbVAR (MECP2)	mailto:dbvar@ncbi.nlm.nih.gov	Lappalainen et al. (2013), Phan et al. (2016)	Database for genomic structural variations, including indels, mobile element insertions, duplications, inversions, translocations, and complex chromosomal arrangements.
http://www.ebi.ac.uk/eva/?Home (European Variation Archive)	mailto:eva-helpdesk@ebi.ac.uk	–	Variant browser. Allows search for variants of specific locations or genes.
http://www.cafevariome.org/	mailto:admin@cafevariome.org	Lancaster et al. (2015)	Meta‐database for genetic variants, genotype‐phenotype databases. Links to 1000 Genomes Project, dbSNP, Diagnostic Variants, Diagnostic Mutation Database, The Frequency of Inherited Disorders Database, Finnish Disease, FORGE Canada Consortium, PhenCode, UniProt, Human Gene Mutation Database, Locus‐specific Databases. Freely available, but some of the linked databases content is only available after registration.
http://www.disgenet.org/web/DisGeNET/menu/home	about:blank	Pinero et al. (2015, 2017)	Database for gene‐disease and variant‐disease associations. Imports data from curated databases like Uniprot, ClinVar, GWAS Catalog, and so on.

### 3.1 Database properties

#### 3.1.1 Aspects of FAIR

In general, the genetic variation or location databases were easier to find than the RTT‐specific ones. Using Google as the search engine for “Rett syndrome database” only RettBase (Christodoulou, Grimm, Maher, & Bennetts, 2003; Krishnaraj, Ho, & Christodoulou, 2017) or excluded databases such as InterRett and the Rett Syndrome Database Network (both of which do not allow direct online access to genotype–phenotype information) were immediately findable—and several publications about RTT databases (e.g., about the Italian Rett database and biobank (Sampieri et al., 2007)). Using more generic terms like “genotype phenotype database” dbGAP (which is an archive for genotype–phenotype studies), DECIPHER and DisGeNET were found. A more specific search result was yielded using meta‐databases for biomedical databases. Seven of the databases were findable on FairSharing.org using the tags “rare disease”, “genetic variation”, or “phenotype”. Others were mentioned in previous publications (Lelieveld et al., 2016) or found through personal recommendation within the scientific community. Considering findability of variants within the database, most offered the possibility to search for variants using at least one of the nomenclatures recommended by the guidelines of the HGVS for genome, RNA or protein changes, or by rs identifiers. The Korean Mutation Database provided no option to search for specific variants, only searches by disease (or disease identifier) were possible. In most cases the databases investigated were publicly accessible; several, however, restricted access to members only (e.g., parts of Café Variome) or were commercial databases with pay to view content (HGMD) (Table 3).

**Table 3:** Description of database structure and information types

Database	↑ Up‐ and↓ Download of dataAPI (if available)	Phenotype information available	Genotype information available
RettBase	↑ Submission of data by mail possible ↓No download function, Web interface No API or similar	Information on whether RTT or not, distinguishes between classical, atypical, preserved speech, and forme fruste RTT, mental retardation (not Rett), Autism	According to HGVS change on the mRNA/cDNA level, RefSeq NM_004992 unless stated otherwise
KMD	↑ Submission of data by registered users ↓No download function, Web interface No API or similar	Diagnosed with RTT using the OMIM identifier (= RTT/RTT preserved speech variant)	According to HGVS change on the mRNA/cDNA level and RefSeq
ClinVar^a^	↑ Possible, detailed submission templates and instructions available ↓ Download/export of search results in form of text files or UI lists possible API available https://www.ncbi.nlm.nih.gov/clinvar/docs/maintenance_use/	Information on whether Pathogenic or not, Diagnosis, for example, RTT, Autism, X‐linked mental retardation	According to HGVS change on the mRNA/cDNA level (mostly) and RefSeq
HGMD “professional”	↑ Not possible, HGMD has its own data acquisition resources ↓ Download and export possible (for registered paying users)	UMLS (ontology) HPO (ontology) OMIM SNOMED CT MeSH	Descriptive: e.g. 11 kb deletion in exon 1–2, HGVS format in the detailed description
LOVD^a^	↑ Upload possible after registration with Submitter clearance ↓ Download of complete database possible, not for specific genes/search results, API available for LOVD 2.0, for LOVD 3.0 http://www.lovd.nl/3.0/docs/manual.html	Variant effect predictor: “+” indicating the variant affects function, “+?” probably affects function, “‐” does not affect function, “‐?” probably does not affect function, “?” effect unknown, “.” effect not classified.	According to HGVS change on the mRNA/cDNA, DNA and protein level and RefSeq
DECIPHER	↑Open upload, bulk upload templates ↓ Web interface, and “Anonymised consented DECIPHER data can be made available in the form of a downloadable encrypted file from a secure server under a data access agreement. Please see the section on data access agreement on the Data Sharing page.” API available https://support.focusvision.com/Decipher/REST_API/010_Decipher_REST_API_2.0	Detailed phenotype description, using HPO annotations	According to HGVS change on the mRNA/cDNA level and Ensemble ID of transcript used (includes RefSeq)
EVS	↑ Data is exclusively from NHLBI GO Exome Sequencing Project (ESP) ↓ Bulk download files, download of specific gene variant information search results as text or VCF No API or similar	Variant effect prediction by PolyPhen2	According to HGVS on the mRNA/cDNA and protein level, rs IDs
ExAC Browser	↑ No upload possible, ExAC includes data from a list of projects ↓ Export of variation table as CSV possible API available http://exac.hms.harvard.edu	Variant effect prediction: Consequence of variation, for example, intronic variation, and consequence of protein aa change	rs IDs, genomic position, RefSeq and allele
dbSNP^a^	↑ Submission possible either directly or via EVA, dbGAP or ClinVar ↓ Possible, “Send to file” function for search results, batch query function for machine readability API at https://www.ncbi.nlm.nih.gov/variation/tools/reporter/docs/api/perl	Variant effect prediction, consequences like, for example, intronic variation, and consequence of phenotype, for example, increased susceptibility to diseases, is given. No RTT mutations are yet available.	rs IDs, HGVS (mRNA/cDNA)
dbVAR^a^	↑ Possible, no clinical data (ClinVar), no sensitive data (dbGAP) ↓”Send‐to‐file” function API at https://www.ncbi.nlm.nih.gov/variation/tools/reporter/docs/api/perl	Clinical Assertion: pathogenic/uncertain significance	rs ID and allele
EVA^a^	↑ Open to everyone, submission guidelines ↓ Free ‐ Export function (CSV), API available http://www.ebi.ac.uk/eva/?API	Variant effect prediction by PolyPhen2/SIFT	rs IDs and allele
Cafe Variome	↑ Upload direct to Café Variome “hosted” or “in‐a‐box” ↓ Export of search results in different formats (CSV, html, LOVD…) API available http://demo2.cafevariome.org/docs/api	dbSNP: “phenotype” column, no entries HGMD: no phenotype data Locus specific: no phenotype data PhenCode: phenotype entry for 1/5 of entries: Diagnosis (RTT, X‐linked mental retardation) Uniprot: same as PhenCode	dbSNP: HGVS (mRNA/cDNA) allele and RefSeq, HGMD: no variant data visible Locus specific: HGVS (mRNA/cDNA) allele and RefSeq PhenCode: HGVS (mRNA/cDNA), Reference links to original data source, Uniprot: HGVS (mRNA/cDNA), reference links to UniProt ID
DisGeNET^a^	↑ No submission, adding of data by text mining and other databases ↓Download of search results possible in different formats (download page http://www.disgenet.org/web/DisGeNET/menu/downloads), provides a SPARQL endpoint	Diagnosis	rs IDs

a Findable at FairSharing.org.

FAIRness, for human users, was hindered by a variety of factors. For example, while many databases provided a search function, one of the core aspects of “F”—that data records are uniquely identified—was frequently overlooked by providers. Often, there was found to be a preference for embedded javascript/AJAX “reveals” of otherwise unidentified data, and/or incremental drill‐down searches until only one result remained. Furthermore, impediments to the “I” and “R” elements of FAIRness—Interoperability and Reusability—were evident in the sparse use of ontological terms, use of ontological terms without indicating their source ontology, and lack of easy‐to‐find citation information for individual data points within aggregate data. On the positive side of FAIRness for humans, however, the terms of data access and re‐use, for example, licensing and use for further studies, were reasonably well implemented in most databases. Not all data could be accessed and reused but the terms and conditions of use were clearly presented and a contact person or consortium was given.

FAIRness for machines was not evaluated, as, in most cases, the data providers made little or no effort to support automated accessibility or interoperability. The notable exception was DisGeNET, with its adoption of nanopublications (data structures that link data, data‐provenance and citation‐related information in a manner that can easily be interpreted by machines (Mons et al., 2011)), and provision of a SPARQL (SPARQL Protocol and RDF Query Language) query interface for these nanopublications (Fu et al., 2015). Where available, a link to each database's API is given in Table 3.

#### 3.1.2 Up and download functionality

It was possible to download or export search results as txt, CSV, RDF, XML, or other formats in ClinVar, EVS, EVA, ExAC, Café Variome, dbSNP, dbVAR, and DisGeNET (Table 3). For DECIPHER, the exporting of data to a file was possible upon request, and in HGMD for paying users. Several databases were found to encourage and accept data submission and provide upload functions or submission contacts. However, others were more restricted in this. For example, DisGeNET retrieves data from other (curated) databases and does not allow direct upload, EVS and ExAC have a defined list of sources (e.g., projects) from which the data is provided, and HGMD has its own data retrieval pipeline.

#### 3.1.3 Genotype and phenotype information format

Currently, there are two major forms in which genetic variants are given in databases: HGVS nomenclature and rs identifier. Four databases give only the rs ID (three of them include the respective allele), seven (including all Café Variome entries) only HGVS, and two both (Table 3).

The extent to which phenotype information is given was found to vary between the different databases (Table 3). Generally, there is a distinction between diagnosis‐based information (six databases out of 13), phenotype (two, including HPO annotation), and predicted pathogenicity scores (PolyPhen/SIFT) (six). For example, ClinVar and PhenCode (PhenCode available via Café Variome) give the clinical diagnosis (e.g., RTT) including variants (e.g., RTT, preserved speech variant) while genetic variant databases provide other information. For example, LOVD, shows whether a variant is pathogenic (severe (+/+) or minor (−/+)) or not (−/−) based on the PolyPhen score; this is also the case in EVS. DECIPHER and HGMD provide detailed phenotypic information which is properly annotated using an ontology (HPO). HGMD in fact provides several options (diagnosis and phenotype). With regard to the RTT‐specific databases, a search of RettBase yielded only information on the diagnosis (with variants), but no details about the associated phenotype (e.g., epilepsy, scoliosis, medication).

### 3.2 RTT‐specific information

#### 3.2.1 Total number of *MECP2* entries

The greatest number was *MECP2* entries were found in RettBase (4738) (Table 4). dbSNP and LOVD both offer around 4500 entries (4229 and 4588), ClinVar 1145. Most other databases offer a few hundred *MECP2* entries. EVS, EVA, dbSNP, dbVAR, ClinVar, and the ExAC Browser exchange information. DisGeNET imports information from ClinVar, so provides nothing new (Table 2).

**Table 4:** Number of database entries for *MECP2* or RTT in general and five specific variants (status March 2018). Number: variant present in this number, + variant present, displayed without details, − variant not found

Database	Total number of MECP2 variant entries	Variant 1 c.397C>T missense	Variant 2 c.502C>T nonsense	Variant 3 c.916C>T missense	Variant 4 c.1200_1222del	Variant 5 c.765_1350del
RettBase	4738 (897 unique)	217	363	246	−	−
Korean Mutation Database	35	1	1	1	−	−
ClinVar	1145	1	13	13	−	−
HGMD “professional”^a^	975	−	+	+	−	+
LOVD3.0 MECP2	4588 (807 unique)	197	335	218	−	+
DECIPHER	203	6	4	2	−	−
EVS	117	−	−	−	−	−
ExAC	599	−	−	−	−	−
dbSNP^a^^,^ b ^,^ c	4229	+	+	+	−	−
dbVAR^c^	469	+	+	+	−	−
EVA	378	+	+	+	−	−
Cafe Variome – dbSNP^a^	500	−	1	1	−	−
Cafe Variome – PhenCode	809	1	1	1	−	−
Cafe Variome – UniProt	71	1	−	1	−	−
Cafe Variome – HGMD^a^	249	−	−	−	−	−
Cafe Variome – Locus‐specific Databases	10	−	−	1	−	−
DisGeNET^b^	+	+	+	+	−	−

a dbSNP and the Café Variome request to dbSNP provided different numbers for MECP2 entries, the same applies for LOVD and HGMD. As the Café Variome link uses the public version of HGMD the exact variants are not shown.

b Search was done via rs number which does not give the exact variation, only position.

c The numbers for dbSNP and dbVAR are from NCBI's Variation Viewer for *MECP2* (GRCh37.p13).

#### 3.2.2 Availability of the five test variants

We used the mutations listed in Table 1 to perform a test search in the selected databases. The first three mutations, which are well known, and in literature well‐described mutations (c.397C>T, c.502C>T, and c.916C>T) were found most abundantly, with over 400 entries in almost all databases. The fourth (c.1200_1222del) was not found at all, and the fifth (c.765_1350del) was found only twice, in LOVD (*MECP2* gene homepage) and HGMD. These last two are derived from NGS studies indicating that the data submission pipelines of this data to genotype‐phenotype databases are not yet that well established.

## 4 DISCUSSION

In this study, we surveyed currently available genotype–phenotype databases using *MECP2* variants in RTT as a test case. We assessed the database structures and functionality and gave an overview of the available data on RTT, *MECP2* variants and their associated phenotypic data, with the aim of enabling data producers and data users to select a database which fits best with their needs to store, look up, and re‐use available data.

### 4.1 Limited availability of *MECP2* gene variants in databases

Our modest inventory of five different *MECP2* variants, of which two were derived from NGS data, underscores the need for further harmonization and integration of gene variant information from different sources. Through a simple survey, we have shown that coverage of five selected variants of the *MECP2* gene in the databases under investigation depends upon both their frequency and how long they have been known. This should not be regarded as a criticism of the individual databases for not containing all possible mutations, but rather as an argument for building a better infrastructure for integration of novel genome sequencing data into databases and improvement of interoperability, similar to that offered by the Beacon project in relation to genomic data. This example of limited coverage in a variety of databases illustrates the fact that despite much progress in NGS, genomic and clinical data are still mainly collected and studied in silos by gene or disease, institution or country. Such a finding is consistent with previous observations (Akle, Chun, Jordan, & Cassa, 2015) and others in the Matchmaker Exchange Special Issue (Human Mutation, Special Issue: The Matchmaker Exchange October 2015, Volume 36, Issue 10, Pages i–iii, 915–1019). It can be explained by several factors, including: regulatory data‐privacy requirements which inhibit secure data sharing across institutions and countries; poor rewarding of people who collect or make individual contributions to data collection; and the incompatibility of file formats and nonstandardized tools and analytical methods (GA4GH, 2016). It is also worth noting that new NGS‐derived variants may often be “hidden” in the published literature, for example, within a cohort identified by a broad diagnosis of intellectual disability (Gilissen et al., 2014), without specific reference being made (e.g., to RTT and/or *MECP*) in the title or abstract of an article. As a consequence, many variants may not be picked up by database curators when trawling the literature for new additions. It is neither the intention nor the recommendation of this article that one database should collect and provide all data but it would be helpful if data could be integrated and findable in such a way that a researcher does not have to search multiple databases to look for one specific variant. In general, adherence to FAIR principles and GA4GH guidelines promise a major improvement.

### 4.2 Need for better sharing of data (interoperability!) within and between RTT‐relevant databases

All of the databases tested in this study are accessible by Web browser (Graphical User Interface, GUI) but not all of them allow download of search results. The lack of a proper API or download function limits data exchange within different databases which leads to the conclusion that the interoperability of these databases is currently rather poor. Making databases interoperable is of particular value as we found that approaches to several databases may be required in order to locate information about a specific mutation and/or to find all of the available phenotypic information. If these databases were generally able to share and exchange data with each other (as some already do, e.g., DisGeNET—ClinVar, RettBase—LOVD), or meta‐databases were available to simultaneously approach several databases through a single search function, the search for information would be much easier.

There is a general problem with multiple entries of the same patients or patient groups. Tracing back the submission to the same author/research group can but may not mean that this is the same patient cohort. As we saw in our database survey, the phenotypic data entry varies greatly, such that multiple entries of the same patient would not automatically be recognized as being the same data. Using data about a patient more than once can lead to statistical bias, especially in the field of rare diseases. For this reason, we would encourage the use of registry identifiers (e.g., ID‐cards) or privacy preserving record linkage (PPRL).

Patient data laws worldwide do not necessarily forbid uploading genetic and phenotypic data to databases (as long as no personal information is also shared), but medical doctors are not always aware of what is permissible, and may opt to “play safe” by not uploading data at all. Information and training for people who actually produce the data (nonbioinformaticians) would, therefore, be helpful. One such example is that started with the ELIXIR training platform (https://www.elixir-europe.org/platforms/training), Bring Your Own Data Workshops (BYOD https://www.dtls.nl/fair-data/byod/), and several other initiatives.

Generally, there is a lack of time and funding to upload and maintain data. Here, we would encourage the community to make mandatory the publishing of datasets alongside the publishing of a research article, as was started with gene‐specific information (see Nucleic Acid Research Instructions to Authors (Walker, Soll, Deutscher, Platt, & Weiner, 1983)) and continued with raw transcriptomics data (journals require upload on databases like GEO or ArrayExpress before publishing), and also to integrate the data in such a way that one study needs to be uploaded only once and is then findable on other platforms (such as http://BioStudies (McEntyre, Sarkans, & Brazma, 2015)). Some positive steps are already being taken in this direction as many European and national grants now require a data management plan for new projects that will allow for sustainability after the project ends.

These problems are not new but were, in fact, flagged up almost 10 years ago when the HVP was initiated (Cotton et al., 2008). At that time, the late Dick Cotton recognized the need to “collect, curate and make accessible information on all genetic variations affecting human health,” and since then, many additional initiatives have been started. To date, the most active and promising of these is the founding of the Global Alliance for Genomics and Health (GA4GH) in 2013. This offers a similar vision and complementary philosophy and approach, with active Working groups and demonstration projects such as the Matchmaker Exchange and Beacon project.

Another issue relates to the knowledge aggregation sites, repositories and in‐house databases which require the owner's agreement to input or download data from the database or grant permission on a case‐by‐case basis when the request to access data is part of a specific research project with prior approval from a medical ethical board. Such databases as the US NHS, Rett Database Network, InterRett, AussieRett, and the Dutch Rett Database (Maastricht) are emerging and there is a need to think about ways to connect them. For a start, each entity must make sure that:

1. their database is populated by relevant and useful data (accurate, up to date), which brings with it implications for data maintenance and sustainability;
2. these data are findable and accessible, which may require reconsideration of their access policy; and,
3. their database provides GUI and API infrastructure for connection with others.

One option could be to use locally installable versions (instances) of genotype‐phenotype databases as offered by LOVD, or PhenomeCentral (Phenotips). These in‐house databases allow collection of patient data and support (ontology) annotations of genetic and phenotype information. Apart from supporting local data collection, exporting and sharing of (non‐patient specific!) meta‐data can be made possible in a second step.

### 4.3 The importance of being FAIR

In our study, we found that, with regard to findability and interoperability of genotype–phenotype databases in particular, there is still much to be done. There are initiatives that work on overcoming this problem. The Beacon project of GA4GH is an initiative that seeks to link molecular data by creating a common searchable infrastructure—the so‐called beacons. At the moment about 70 databases/data‐sources contribute to this. Currently, it is only possible to look for single nucleotide changes—for example, a test search in Beacon for our mutation 1 (X: 153296882 G>A) yielded 13 hits. The search for small, specific insertions/deletions is currently being implemented but is not yet functional for all databases (personal communication). The RD‐connect and Orphanet platforms also provide data—in as much as they link to registries and biobanks, which might have information about the disease. For RTT, the RD‐connect catalogue lists three registries: the Italian National Rare Disease Registry, RaDiCo‐GenIDA, and the Rett Database Network (none of which provide directly available online genetic and phenotypic data and were not, therefore, included in our survey).

### 4.4 The importance of collecting detailed phenotypic information

Among the genetic variants of *MECP2*, there are those that cause RTT, those that cause mild intellectual disability, and there are neutral/benign variants. Among the disease‐causing forms, there are severe and mild variants of typical/classical RTT and atypical RTT, for example, preserved speech variant (Zappella et al., 2001). An underlying minimal set of core and supporting criteria must be fulfilled in order for a clinical diagnosis of RTT to be given (Neul et al., 2010). Despite this, however, both classical and atypical forms display a broad range of phenotypes. To name but a few of the characteristics of the syndrome, some individuals with RTT cannot walk while many do, and most develop scoliosis or epilepsy but not all. Among those with epilepsy, there is no single antiepileptic treatment that works for all, indicating for example, different physiological roots, although practice preferences and availability of specific agents may also affect the choice of medications. Mood and character of individuals vary greatly, too. It is clear that RTT is a complex syndrome with multiple factors—including levels of X‐inactivation, genomic, epigenetic, and other environmental influences—affecting its phenotypic presentation. Currently, there are several approaches to capture the phenotype realized in the databases we investigated:

1. By diagnosis: RTT‐ or disease‐specific databases especially, give the information that the carrier of this *MECP2* mutation has been diagnosed with RTT (or others) (RettBase, ClinVar, Café Variome/PhenCode, DisGeNET, HGMD). In some cases, the diagnosis is even linked to an identifier (OMIM, MeSH, DOID).
2. A detailed description of the phenotype is given—but without diagnosis (DECIPHER, HGMD).
3. The effect of the genetic variation is given as measured/observed or predicted (e.g., using PolyPhen2) molecular biological consequences (LOVD, ExAC, dbSNP) or phenotype “damaging” effect (EVS).

To cover the richness of medical observation, we strongly encourage the collection of detailed phenotype descriptions of genetic variations. One way to contribute to a more detailed elucidation of phenotypes is through encouraging a clearer use of terms which should include the use of ontologies, identifiers and minimal information standards (Lapatas, Stefanidakis, Jimenez, Via, & Schneider, 2015). In this respect, the application of HPO terms is widely advocated within the rare disease field/community, as illustrated by the GA4GH recommendation on this topic (see https://genomicsandhealth.org/working-groups/our-work/phenotype-ontologies). This is where population‐based/epidemiological studies such as the US NHS and AussieRett, both of which track and record the longitudinal natural history of RTT, could make a major contribution in the future.

Finally, we would like to stress two things. First, we recognize that any work such as we are recommending to further develop, maintain and integrate existing databases does not come without costs attached. However, we believe that each of the databases we have investigated in this study is of value and should be well‐supported and well‐funded in order to maximize use of the data and yield maximum long‐term benefits. Second, we recognize that diseases are rarely truly monogenic. All genes function in an environment of other gene products, including their variations (epistasis). In addition to classic examples, such as PKU (Scriver & Waters, 1999) and Cystic Fibrosis (Gallati, 2014), this was recently illustrated in the cancer field with the added value of gene expression data to established oncogenic driver mutations (Voest & Bernards, 2016). A similar argument was put forward by McArthur and colleagues when they advocated for the inclusion of RNA‐seq to increase the diagnostic yield within the field of rare diseases (Cummings et al., 2017). This phenomenon may also be translated to RTT with *MECP2* mutations as major “drivers”. To read and interpret a disease‐causing variant within the individual's genetic environment will be one of the major challenges in the future.
